# Supplementary material for: Learning curve and surgical outcome of robotic assisted colorectal surgery with ERAS program
Source: Sci Rep. 2022 Nov 29;12:20566. doi: 10.1038/s41598-022-24665-w (PMC9709162; doi:10.1038/s41598-022-24665-w)
Supplement: Supplementary file 1 — Supplementary Information 1. [file 41598_2022_24665_MOESM1_ESM.zip › LSC Raw data-Ñ[▒K/066/O_ñw╝╨░O▒Kñσ.pdf]

正式摘要(電子簽章：000000185381503)

W83 / W83

臺中榮民總醫院

Operation Note

[redacted]

- 手術日期：2020/02/07
- 手術醫師：VS 蔣鋒帆，CR 黃佀霖，R 楊涵中，R 邱俊凱
- Anesthesia : GE
- Position : Supine
- Aseptic agents : Soap , Betadine Alcohol

Diagnosis

- Pre-operative diagnosis : Malignant tumor of D-colon
- Post-operative diagnosis : Ditto.

Surgical Indication

- For oncological tumor resection

Operation

- Laparoscopic Anterior resection and anastomosis - malignant
  LEFT OOPHORECTOMY

Finding

- 1. One 3x2 cm Borrmann's type III ulcerative tumor at D-S junction, which invaded through the serosa. Clinical stage: T4N2M0.
  2. There were some enlarged indurated lymph nodes at mesocolon and left branch of middle colic vessels.
  3. There was no visible liver metastasis, no visible peritoneal seeding
  4. Some adhesion between omentum and abdominal wall. Operative methods:
  1. Laparoscopic left hemicolectomy with side-to-side anastomosis, GIA 80/4.8*2, re-inforcement with silk: done.
  2. Vessel ligation: left branch of middle colic and left colic vessels.
  3. Cut margin: proximal 7.5 cm, distal 13 cm.
  4. Reperitonealization: yes.
  5. One 19Fr. JP drain at left subphrenic region.

Procedure

- A Laproscopic anterior resection is done with general anesthesia, with lithotomy position, foley catheter in place. We makes several incision in the lower abdomen to insert the trocar. The position of trocar placement was shoen below. The IMA root was identified and skeleton by harmonic scaple initially. Then IMV was also ligated in the same level. After retroperitoneal dissection , the left side colon and upper rectum was modilized and free from the retroperitoneum, pelvis. The tumor was identified and resected was planned with a safe margin about 10cm in proximal and 5cm in distal. Then we removes the cancer and a margin of normal tissue on either side of the cancer, along with nearby lymph nodes and other tissues around the rectum, by the assisted of endo-GIA 60. the wound around umbilicus was extened to retract the specimen. Then the EEA 29mm was used to reattached to the remaining rectum. After check bleeders mediculously, the penrose drainage tube was placed in the low pelvis. The fascia layer and skin was closed by 1-0 vicryl and 3-0 dermalone. He stood the whole procedure well and sent to POR under stable condition.
- Estimated blood loss: minimum
- 器械數量: 正確
- 紗布數量: 正確

Image

- PAINT
  - colon & rectum

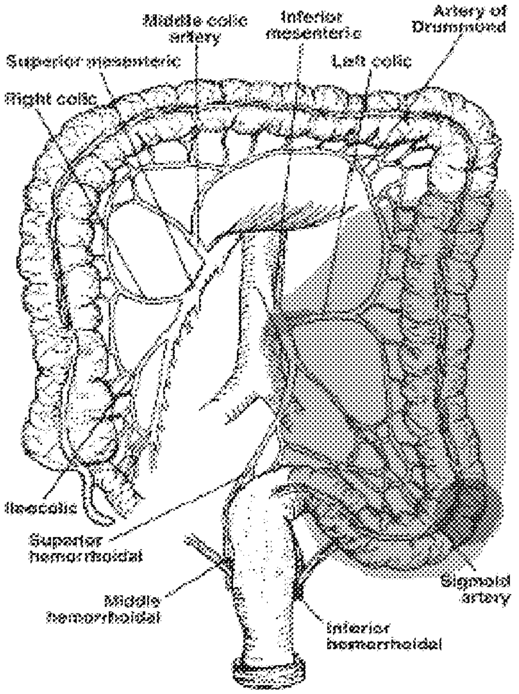

編輯主任 2062F蔣鋒帆、主治 2286H黃俔霖

正式摘要**(電子簽章：000000185381503)**

W83 / W83

doctorRank:B
